# Prognostic value of ascites in patients with liver cirrhosis undergoing cardiac surgery

**DOI:** 10.1186/s13019-023-02393-0

**Published:** 2023-10-28

**Authors:** Amila Cizmic, Parwis Baradaran Rahmanian, Asmae Gassa, Elmar Kuhn, Navid Mader, Thorsten Wahlers

**Affiliations:** 1 Department of General, Visceral and Thoracic Surgery, University Medical Center Hamburg-Eppendorf, Hamburg, Germany; 2 Department of Cardiothoracic Surgery, University Hospital of Cologne, Cologne, Germany

**Keywords:** Ascites, Liver cirrhosis, Cardiac surgery, Postoperative complications, Mortality

## Abstract

**Introduction:**

Mild or moderate liver cirrhosis increases the risk of complications after cardiac surgery. Ascites is the most common complication associated with liver cirrhosis. However, the prognostic value of ascites on postoperative morbidity and mortality after cardiac surgery remains uninvestigated.

**Methods:**

A retrospective study included 69 patients with preoperatively diagnosed liver cirrhosis who underwent cardiac surgery between January 2009 and January 2018 at the Department of Cardiothoracic Surgery, University Hospital of Cologne, Germany. The patients were divided into ascites and non-ascites groups based on preoperatively diagnosed ascites. Thirty-day mortality, postoperative complications, length of stay, and blood transfusions were analyzed postoperatively.

**Results:**

Out of the total of 69 patients, 14 (21%) had preoperatively diagnosed ascites. Ascites group had more postoperative complications such as blood transfusions (packed red blood cells: 78.6% vs. 40.0%, *p* = 0.010; fresh frozen plasma: 57.1% vs. 29.1%, *p* = 0.049), acute kidney injury (78.6% vs. 45.5%, *p* = 0.027), longer ICU stay (8 vs. 3 days, *p* = 0.044) with prolonged mechanical ventilation (57.1% vs. 23.6%, *p* = 0.015) and tracheotomy (28.6% vs. 3.6%, *p* = 0.003). The 30-day mortality rate was significantly higher in the ascites group than in the non-ascites group (35.7% vs. 5.5%, *p* = 0.002).

**Conclusion:**

Ascites should be implemented in preoperative risk score assessments in cirrhotic patients undergoing cardiac surgery. Preoperative treatment of ascites could reduce the negative impact of ascites on postoperative complications after cardiac surgery. However, this needs to be thoroughly investigated in prospective randomized clinical trials.

**Graphical abstract:**

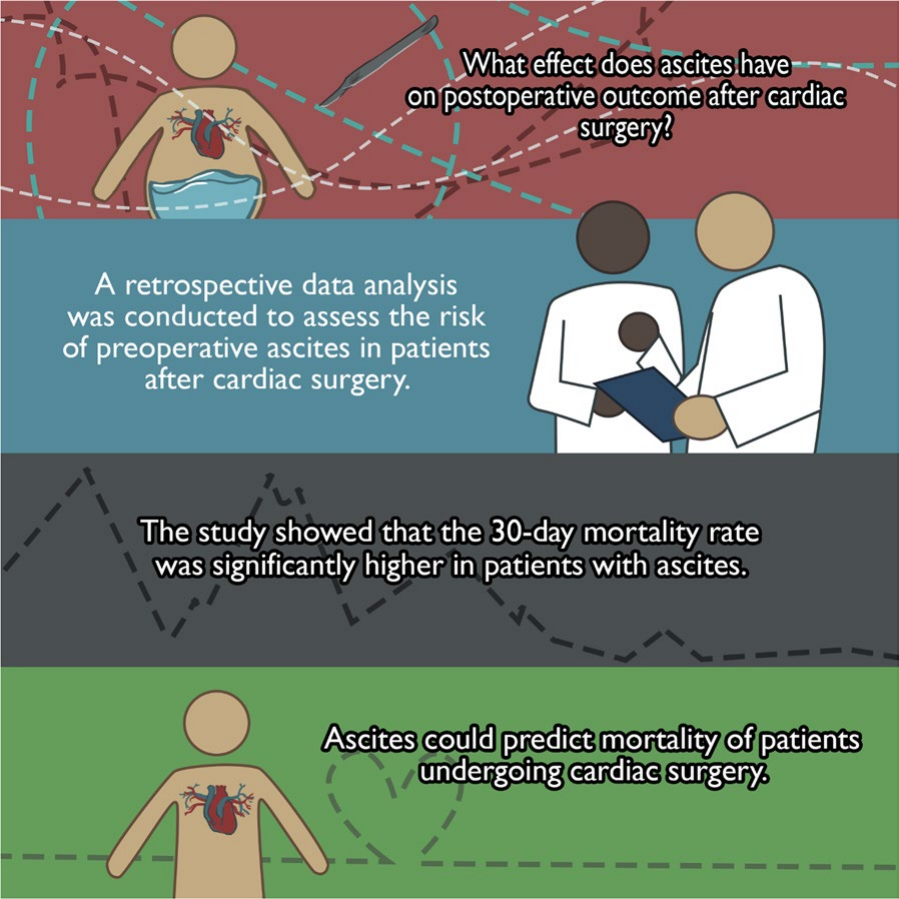

## Introduction

Open heart surgery in patients with liver cirrhosis (LC) is associated with considerable risks owing to cirrhosis-related coagulopathy, enhanced risk of infection, and a potential anoxic liver injury due to cardiopulmonary bypass (CPB) [1].

Along with the MELD score, the severity of liver cirrhosis is mainly classified by the Child-Turcotte-Pugh (CTP) criteria, based on the presence of encephalopathy, the severity of ascites, total bilirubin level, albumin level, and prothrombin time or international normalized ratio (INR) [2]. Since these variables are regularly acquired with minimally invasive methods in everyday clinical practice, the CTP score has become a broadly accepted method for determining liver function and its influences on the postoperative risk status [3, 4]. However, LC has not been marked as an independent preoperative risk factor by routinely used EuroSCORE, and its impact on postoperative outcomes remains unclear [5–7]. On the other hand, the Society of Thoracic Surgeons (STS) preoperative risk score assessment includes liver disease as a risk factor for mortality after cardiac surgery. Furthermore, several new STS data variables, including liver disease, were significantly correlated with operative mortality after cardiac surgery [8].

It has been reported that mild or moderate LC increases complications after elective cardiac surgery, increasing the Intensive Care Unit (ICU) and overall hospitalization time [9]. Various factors attributed to LC rather than cardiac surgery could be responsible for the poor postoperative prognosis. These factors, such as ascites, thrombocytopenia, compromised immune system, and gastrointestinal disorders, correlate with LC severity [10]. However, no definite prognostic factors have been identified for cirrhotic patients undergoing cardiac surgery because the number of patients and studies remain small [11].

Ascites is defined as an accumulation of more than 25 ml of fluid in the peritoneal cavity [12]. It is caused by renal sodium retention due to increased renin–angiotensin–aldosterone system response to the splanchnic circulation's vasodilation. It is the most common complication associated with cirrhosis, increased mortality, morbidity, and poor long-term outcomes [12]. The aim of this retrospective data analysis was to establish the prognostic value of ascites in predicting postoperative outcomes in cirrhotic patients undergoing cardiac surgery.

## Materials and methods

We performed a retrospective single-center data analysis of cirrhotic patients undergoing cardiac surgery between January 2009 and January 2018 at the Department of Cardiothoracic Surgery, University Hospital of Cologne, Germany. The Ethics Committee of the University of Cologne waived its consent (EK 21-1471-retro).

We included 69 patients with preoperatively diagnosed LC by clinical data, radiological findings (abdominal computed tomography, magnetic resonance imaging, or ultrasonography), or liver biopsy when applicable.

Demographic characteristics, baseline laboratory values, and comorbidities were extracted from the institutional database. We analyzed the data by looking at 30-day mortality, postoperative complications, length of ICU and total in hospital stay, and blood transfusions.

CTP score was calculated using the following variables: ascites (absent, mild, moderate), serum albumin (mg/dL), INR, total bilirubin (mg/dL), and severity of hepatic encephalopathy (grade 1–3). The CTP classification was defined based on the resulting score: (A: 5–6, B: 7–9, and C: 10–15 points) [13]. Ascites was diagnosed preoperatively either during the clinical examination or using ultrasound. Ascites was defined as an accumulation of fluid in the peritoneal cavity. None of the patients had preoperative treatment (fluid/salt restriction and/or interventional relief of the intraabdominal fluid) of the ascites on admission to the surgical department. The EuroScore II was calculated using the official EuroScore II website calculator (https://www.euroscore.org/index.php?id=17). Preoperative diagnosis of portal hypertension was retrieved from the medical documentation of the patients prior to admission to the surgical department. Acute kidney injury (AKI) was defined according to Kidney Disease: Improving Global Outcomes (KDIGO) standards [14]. Prolonged mechanical ventilation was defined as invasive mechanical ventilation longer than 72 h after the surgery. Anemia was defined as hemoglobin values less than 13.5 g/dl in male or less than 12.0 g/dl in female patients. Thrombocytopenia was defined as a blood platelet count less than 150.000/microliter.

Statistical analyses were performed using IBM SPSS Statistics version 26 (IBM Corp, Armonk, New York, United States). Patient-relevant descriptive statistics are presented with percentages for categorical variables and standard deviations for continuous variables. In addition, univariate and multivariate logistic regression was performed to estimate odds ratios with 95% confidence intervals to determine 30-day mortality predictors in cirrhotic patients undergoing cardiac surgery. All reported *p* values are two-sided, and *p* values of < 0.05 were considered statistically significant.

## Results

### Patient characteristics

Out of 16 772 cardiac surgeries performed between January 2009 and January 2018 at the Department of Cardiothoracic Surgery, University Hospital of Cologne, Germany, we identified 69 (0.41%) patients with LC who underwent different types of cardiac surgery. Patients were divided into two groups:, a non-ascites group [55 patients (79.7%)] and an ascites [14 patients (20.3%)] group. All demographic characteristics, preoperative risk factors of the patients and surgical parameters are shown in Table 1. Patients with ascites had higher body weight (84.5 ± 13.8 vs. 81.0 ± 19.4 kg, *p* = 0.022) and more often anemia (71.4% vs. 36.4%, *p* = 0.018) preoperatively than the ones without ascites. The preoperative risk scores (STS and EuroScore II) showed no significant differences between the two groups. Other demographic features and comorbidities did not differ between the two groups (Table 1).

**Table 1:** Preoperative and surgical parameters of the patients

Parameter	Ascites group n = 14 (20.3%)	Non-Ascites group n = 55 (79.7%)	*p* value
Age (years)	65.9 ± 8.7	68.1 ± 9.2	0.736
Male gender	11 (78.6%)	38 (69.1%)	0.485
Height (cm)	174.6 ± 9.0	172.4 ± 8.5	0.344
Weight (kg)	84.5 ± 13.8	81.0 ± 19.4	0.022
BMI (kg/m^2^)	27.7 ± 4.3	27.2 ± 6.1	0.311
STS	4.7 ± 2.4	4.8 ± 2.6	0.282
EuroScore II	5.8 ± 4.8	5.3 ± 5.6	0.518
DM Type II	9 (64.3%)	24 (43.6%)	0.167
CKD	6 (42.9%)	30 (54.5%)	0.643
Dialysis	1 (7.1%)	2 (3.6%)	0.566
PAD	3 (21.4%)	10 (18.2%)	0.781
AF	5 (35.7%)	19 (34.5%)	0.935
AV block	1 (7.1%)	3 (5.5%)	0.809
Active smoking	6 (42.9%)	26 (47.3%)	0.767
CTP classification			0.646
A	7 (50.0%)	35 (63.6%)	
B	6 (42.9%)	17 (30.9%)	
C	1 (7.1%)	3 (5.5%)	
Hepatitis	1 (7.1%)	7 (12.7%)	0.573
B	1 (7.1%)	3 (5.5%)	
C	0 (0%)	4 (7.3%)	
Active alcohol consumption	6 (42.9%)	33 (60.0%)	0.248
Portal hypertension	6 (42.9%)	20 (36.4%)	0.654
Splenomegaly	3 (21.4%)	17 (30.9%)	0.485
Hemorrhagic diathesis	7 (50.0%)	32 (58.2%)	0.581
Esophageal varices	7 (50.0%)	21 (38.2%)	0.421
Encephalopathy	2 (14.3%)	2 (3.6%)	0.128
Anemia	10 (71.4%)	20 (36.4%)	0.018
Thrombocytopenia	7 (50.0%)	28 (50.9%)	0.952
Marcumar	3 (21.4%)	12 (22.8%)	0.975
Surgical parameters			
ACVB	9 (64.3%)	17 (30.9%)	0.021
Valve surgery	2 (14.3%)	30 (54.5%)	0.007
Combined ACVB and valve surgery	3 (21.4%)	8 (14.5%)	0.530
Urgent surgery	8 (57.1%)	18 (32.7%)	0.092
Previous cardiac surgery	2 (14.3%)	6 (10.9%)	0.725

BMI, body mass index; DM, diabetes mellitus; CKD, chronic kidney disease; PAD, peripheral arterial disease; AF, atrial fibrillation; AV, atrioventricular; CTP, Child-Turcotte-Pugh; ACVB, aortocoronary venous bypass. Data are presented as number (percentage) for categorical variables, mean standard deviation ± for normally distributed or median, and [25th und 75th percentile] for not normally distributed continuous variables. Accordingly, Chi-Quadrat, exact Fisher, Student’s t-test, or Mann–Whitney U test were used to compare

### Postoperative complications and mortality

Patients with ascites needed more packed red blood cells (pRBC) and fresh frozen plasma (FFP) transfusions than the non-ascites patients (pRBC: 78.6% vs. 40.0%, *p* = 0.010; FFP: 57.1% vs. 29.1%, *p* = 0.049). Further postoperative complications are shown in Table 2.

**Table 2:** Comparison of postoperative complications and 30-day mortality between the two groups

Parameter	Ascites group n = 14 (20.3%)	Non-ascites group n = 55 (79.7%)	*p* value
CPR	1 (7.1%)	3 (5.5%)	0.758
Rethoracotomy	3 (21.4%)	7 (12.7%)	0.409
Pericardial tamponade	2 (14.3%)	4 (7.3%)	0.406
ECMO	0 (0%)	1 (1.8%)	0.611
IABP	1 (7.1%)	4 (7.3%)	0.987
GI bleeding	1 (7.1%)	2 (3.6%)	0.566
Postoperative delirium	4 (28.6%)	9 (16.7%)	0.313
Delayed recovery from anesthesia	6 (42.9%)	14 (26%)	0.200
Prolonged mechanical ventilation	8 (57.1%)	13 (23.6%)	0.015
Tracheotomy	4 (28.6%)	2 (3.6%)	0.003
Nosocomial sepsis	2 (14.3%)	9 (16.7%)	0.850
Wound infection	4 (28.6%)	11 (20.0%)	0.488
AKI	11 (78.6%)	25 (45.5%)	0.027
Postoperative dialysis	4 (28.6%)	7 (12.7%)	0.148
Postoperative AF	1 (7.1%)	6 (10.9%)	0.677
Postoperative pacemaker implantation	0 (0%)	2 (3.6%)	0.469
Blood transfusion			
pRBC > 5 packs	11 (78.6%)	22 (40.0%)	0.010
FFP > 4 packs	8 (57.1%)	16 (29.1%)	0.049
Platelet > 1 pack	11 (78.6%)	28 (50.9%)	0.062
In hospital stay (days)	20 [6;212]	13 [1;70]	0.118
ICU stay (days)	8 [1;210]	3 [1;70]	0.044
30-day mortality	5 (35.7%)	3 (5.5%)	0.002

CPR, cardiopulmonary resuscitation; ECMO, extracorporeal membrane oxygenation; IABP, intra-aortic balloon pump; GI, gastrointestinal; AKI, acute kidney injury; AF, atrial fibrillation; pRBC, packed red blood cell; FFP, fresh frozen plasma; ICU, intensive care unit. Data are presented as number (percentage) for categorical variables, mean standard deviation ± for normally distributed or median, and [25th and 75th percentile] for not normally distributed continuous variables. Accordingly, Chi-Quadrat, exact Fisher, Student’s t-test, or Mann–Whitney U test were used to compare

AKI occurred more often in patients with ascites than in patients without ascites (78.6% vs. 45.5%, *p* = 0.027), with no significant difference in the rate of new postprocedural dialysis between the two groups (28.6% vs. 12.7%, *p* = 0.148).

Ascites patients stayed longer in the ICU than those without ascites, with a median of 8 and 3 days (*p* = 0.044).

Prolonged mechanical ventilation and tracheotomy rates were more often observed in ascites than in non-ascites patients (57.1% vs. 23.6%, *p* = 0.015; 28.6% vs. 3.6%, *p* = 0.003, respectively).

The 30-day mortality was significantly higher in the ascites group than in the non-ascites group (35.7% vs. 5.5%, *p* = 0.002). Three out of five deceased patients with ascites developed acute liver failure and bowel ischemia with septic shock. The remaining two patients with ascites died after the surgery due to combined septic and cardiogenic shock. One patient in the non-ascites group died due to bowel ischemia. The remaining two patients had multiorgan failure owing to cardiogenic and septic shock.

Several preoperative factors were compared by multivariate logistic regression analysis to determine possible risk factors contributing to postoperative mortality (Table 3). Ascites was the only preoperative risk factor associated with increased 30-day mortality (odds ratio 9.63; 95% confidence intervals 1.95–47.54).

**Table 3:** Multivariate analysis of 30-day mortality

Parameter	Adjusted OR (95% CI)	*p* value
Age (years)	1.82 (0.47–2.96)	0.721
STS	1.13 (0.75–1.69)	0.558
EuroScore II	1.01 (0.85–1-19)	0.946
DM Type II	1.68 /0.29–9.57)	0.562
CKD	2.06 (0.95–4.24)	0.374
PAD	1.05 (0.14–7.67)	0.958
AF	0.57 (0.10–3.30)	0.548
Active smoking	5.07 (0.52–49.07	0.161
Alcohol consumption	2.06 (0.25–16.84)	0.499
Ascites	9.63 (1.95–47.54)	0.005
Portal hypertension	5.53 (0.39–77.44)	0.206
Splenomegaly	3.94 (0.41–37.35)	0.232
Esophageal varices	2.67 (0.47–152.74)	0.634
Encephalopathy	6.86 (0.36–128.52)	0.197
Anemia	15.99 (0.81–258.49)	0.458
Thrombocytopenia	0.31 (0.33–2.85)	0.301

OR, odds ratio; CI, confidence interval; DM, diabetes mellitus; CKD, chronic kidney disease; PAD, peripheral arterial disease; AF, atrial fibrillation

Other postoperative complications were not different between the two groups.

## Discussion

This retrospective data analysis assessed a group of patients with LC who underwent various cardiac surgeries, emphasizing the differences between patients with and without ascites regarding the postoperative complications and 30-day mortality. Cirrhotic patients with ascites developed more often postoperative complications and had higher 30-day mortality rate than cirrhotic patients without preoperative ascites after a cardiac surgery. Ascites is known as the most common complication of LC, and its development indicates a poor prognosis [15, 16

In the presented data analysis, patients with ascites did not suffer more from comorbidities than patients without ascites. Anemia was the only preoperative parameter observed more often in ascites than in the non-ascites group. Anemia was found to be an independent predictor for the development of acute-on-chronic liver failure and is associated with increased mortality [17]. The degree of hepatic dysfunction and portal hypertension is described to correlate with the severity of anemia. It is reported that the incidence of ascites was also higher in cirrhotic patients with anemia than in those without [18]. However, the CTP classification in the presented study did not show any significant difference between the two groups, indicating a similar distribution of the stage of the liver disease in both groups and not entirely supporting the role of liver dysfunction in developing anemia. However, the presence of the preoperative anemia could have influenced the higher postoperative 30-day mortality in the ascites group.

Even nowadays, cardiac surgery is still burdened by a high rate of postoperative pulmonary complications, up to 25% [19]. There was a significantly greater need for prolonged mechanical ventilation and tracheotomy in patients with than in those without ascites (57.1% vs. 23.6%, *p* = 0.015; 28.6% vs. 3.6%, *p* = 0.003). It has been described that ascites and fluid overload may cause or worsen pulmonary function due to atelectasis and pulmonary edema. The end-expiratory lung volume can be decreased, impairing lung and chest wall function and gas exchange [20]. This and the known risks of pulmonary complications after cardiac surgery may explain the influence of ascites on the need for prolonged mechanical ventilation, which was, in some cases, followed by tracheotomy in patients with preoperatively diagnosed ascites.

On further postoperative complications, ascites patients developed AKI more often than non-ascites patients. AKI is a frequently present complication after cardiac surgery with a strong influence on morbidity and mortality [21]. The role of ascites in the development of AKI in cirrhotic patients has already been described. LC can lead to renal dysfunction and hepato-renal syndrome, which occurs in conjunction with microcirculatory dysfunction in other organs, including the heart and the peripheral vascular bed [22]. Therefore, patients with ascites are at a higher risk of developing progressive renal impairment [16], adding to the known procedural cardiac surgery risk in AKI occurrence.

Postoperative bleeding complications followed by an excessive need for pRBC and FFP transfusions were significantly more often observed in the ascites than in the non-ascites group. Patients with chronic liver disease show a decline in platelets' number and function, making them more prone to bleeding complications [23]. Because most coagulation factors are synthesized in the liver, liver damage can easily lead to abnormal coagulation or a tendency to bleed profusely [24]. With usual systemic heparinization during cardiac surgery, these factors could lead to postoperative bleeding in this specific patient group. In addition, portal hypertension goes side to side with the occurrence of ascites and may lead to congestive splenomegaly with the trapping of platelets and thrombocytopenia, resulting in further postoperative bleeding [25, 26]. However, it is important to mention that the ascites group had more anemic patients than the non-ascites group, which could be a potential reason for increased pRBC transfusion. Nevertheless, this does not explain the increased FFP transfusion rate in the ascites patients.

Furthermore, since the degree of portal hypertension was not routinely assessed in this cohort of cardiac surgery patients, we could not analyze its influence on the outcomes more thoroughly.

It is known that long-term survival after cardiac surgery in patients with LC is lower compared to the overall population [3]. The development of ascites in LC predicts a poor prognosis with a 50% mortality chance within three years [27]. Ascites patients in this retrospective data analysis had higher postoperative 30-day mortality than non-ascites patients (35.7% vs. 5.5%, *p* = 0.002). The analysis of cirrhotic patients' postoperative outcomes after cardiac surgery has been limited to small, single-institutional studies and have, therefore, a rather narrow interpretation. However, Singh et al. examined the complication rates in more than two thousand cirrhotic patients after ACVB surgery and reported that ascites was associated with increased in-hospital morbidity and mortality. Similarly, Shaheen et al. described ascites as a predictor of mortality in patients with liver cirrhosis after ACVB surgery (odds ratio 3.80; 95% confidence intervals of 1.95–7.39). These results support the presented findings of the increased postoperative complications and 30-day mortality in this single-centre retrospective data analysis. It is important to mention, that the ascites group had more urgent surgeries than the non-ascites group, which could have contributed to the higher 30-day mortality rate.

Despite the small sample size, we investigated the potential predictive value of ascites on postoperative mortality. The odds ratio was 9.63 with 95% confidence intervals of 1.95–47.54. Thus, it shows that ascites could potentially be a predictive factor of postoperative mortality in cirrhotic patients with preoperative ascites undergoing cardiac surgery.

This study is limited by its retrospective design, a small group of patients with preoperative ascites, and the fact that it is performed on a single-center data registry. The potential multifactorial cause of ascites (hepatorenal syndrome and/or heart insufficiency) could also influence the outcomes of the study. Furthermore, 30-day mortality cannot be sufficiently evaluated in uni- and multivariable logistic regression analysis, which could further support ascites' prognostic value and quantify covariates' impact. Finally, the calculated odds ratio for predicting postoperative mortality should be interpreted cautiously due to the small sample size.

## Conclusion

Ascites could predict morbidity and 30-day mortality in patients with liver disease undergoing cardiac surgery and should be implemented in preoperative risk score assessments. Preoperative treatment of ascites with reduced salt intake, diuretic medication, or paracentesis could reduce ascites' negative impact on postoperative complications and mortality. However, this hypothesis must be thoroughly investigated in future randomized clinical trials.

## Data Availability

The datasets used and analyzed during the current study are available from the corresponding author on reasonable request.

## Abbreviations

AF: Atrial fibrillation
AKI: Acute kidney injury
AV: Atrioventricular
BMI: Body mass index
CKD: Chronic kidney disease
CPB: Cardiopulmonary bypass
CPR: Cardiopulmonary resuscitation
CTP: Child-Turcotte-Pugh
DM: Diabetes mellitus
ECMO: Extracorporeal membrane oxygenation
FFP: Fresh frozen plasma
GI: Gastrointestinal
IABP: Intra-aortic balloon pump
ICU: Intensive care unit
INR: International normalized ratio
KDIGO: Kidney disease: improving global outcomes
LC: Liver cirrhosis
PAD: Peripheral arterial disease
pRBC: Packed red blood cells
STS: Society of thoracic surgeons
